# Online Small Sample Learner Modeling and Curriculum Recommendation with Healthy Emotional Factors of College Students

**DOI:** 10.1155/2022/8247203

**Published:** 2022-02-27

**Authors:** Xia Li, Ruibo Di, Jun Cai

**Affiliations:** Zhejiang Gongshang University, Hangzhou College of Commerce, Hangzhou, Zhejiang 311599, China

## Abstract

Online education is a popular way for college students at present, and it is also a good compensation way to meet the special situation that traditional offline teaching cannot complete the teaching task. Traditional classroom teaching methods have been difficult to meet the learning requirements of contemporary college students, while online classroom has made up for the shortcomings of traditional classroom teaching to some extent because of its short class hours, prominent focus, and online mobile learning. First, this paper proposes an online SSL (small sample learner) model for college students to integrate healthy emotional factors. The characteristics of learners are divided into three categories: basic characteristics of learners, characteristics of behavioral factors, and characteristics of emotional factors, and the problem of solving mapping functions is transformed into the problem of solving kernel functions. Second, a novel curriculum recommendation model integrating healthy emotional factors is proposed, which fully considers the influence of user comments on similarity and transforms the similarity of users in the overall score of the project into the similarity of users in the emotional tendency of the special attributes of the project. Through the experimental evaluation, the accuracy and stability of the recommendation are greatly improved.

## 1. Introduction

With the continuous deep integration of big data, the Internet of Things, cloud computing, and other information technologies and education fields, the intelligent transformation of education has been promoted, and the advanced educational form in the era of educational informationization 2.0 represented by intelligent education has been achieved [1, 2]. At present, there are still some technical problems in intelligent education in the aspects of learning guidance, recommendation, answering questions, and evaluation, such as how to eliminate the semantic gap between learners and resources in resource recommendation; how to realize the large-scale online learners' answering guidance [3]; how to carry out refined teaching evaluation on learners, teachers, learning environment, and other objects.

Learning and communication through the online learning community provide people with a generalized learning method without the limitation of the number of participants and time and place [4]. Learning activities in such an environment can make everyone enjoy high-quality teaching resources and fair learning opportunities. Learning behavior data of learners in online learning communities can complete modeling and analysis of learners' behaviors, which can promote teachers and community managers to be more professional and meticulous in teaching management. A large amount of data has promoted the prosperity and development of artificial intelligence in recent years, especially the direction of DL (deep learning) [5, 6]. Due to its extremely nonlinear characteristics, DL can learn hidden patterns from a large amount of data. On the other hand, the rapid development of artificial intelligence in recent years largely depends on the development of data. Using learning data for modeling and analysis can help them to improve the quality of community content and various functions and inspire new ideas: from the perspective of teaching mode reform [7], by studying learners' learning behaviors, and can predict their learning trends, which can help them further in the rapid development of educational informationization.

Building a user portrait system can accurately depict each learner's personalized characteristics and then create a targeted study plan based on the subtle differences between different learners, which includes recommending currently suitable subjects based on learners' background knowledge structure, arranging course schedules based on learners' learning ability, recommending relevant counterpart courses based on learners' interests and hobbies, and predicting a learner's future success. Based on a review of the literature on learner models, this paper focuses on the recognition of learners' facial expressions and methods for obtaining emotional data and builds an online SSL (small sample learner) learner model that incorporates healthy emotional factors in an online classroom setting. Following that, a quantitative analysis method of curriculum recommendation model [8] suitable for integrating healthy emotional factors is discovered through an in-depth study of quantitative analysis technology of learners' characteristics.

## 2. Related Work

In the analysis and mining of educational big data, related universities have done a series of research and applications. For example, literature [9] developed an online learning assistant based on learner behavior analysis and intelligent dialogue. Taking the learning subject as the object, the key technologies such as the construction of an intelligent teaching environment, educational measurement, and evaluation are studied. Literature [10] constructs a learner behavior model by extracting the characteristics of learners' course completion rate and dropout rate and then predicts learners' learning behavior and judges whether learners can finally complete the course learning. Literature [11] studies the learning track and behavior data of college students learning Russian on an online education platform, extracts the data set of behavior characteristics for clustering analysis, and classifies learners according to the clustering results, so as to complete the special recommendation for learners. The literature [12] proposes a decision tree-based user performance prediction model. The end result reveals that the majority of students are in a passive learning mode. It also demonstrates how to use data mining technology to improve online teaching and learning, as well as making recommendations for students. Literature [13] proposes a comprehensive cognitive model for reconstruction analysis that integrates learners' comprehensive factors. A multimedia cognitive model has been proposed in the literature [14]. Literature [15] investigates the relationship between Facebook photos and users' personalities, primarily through Facebook's basic visual feature coding, which associates the activities associated with users' photos with the five-factor personality model. According to the literature [16], learners' utterances contain a great deal of information about their personalities, feelings, opinions, and habits in addition to semantic content.

FSL is an important goal of researchers in the process of exploring intelligent systems. Most researches on FSL are focused on neural networks [16, 17]. Literature [18] proposes to use a hierarchical nonparametric Bayesian model to realize FSL. In this model, categories are represented as tree-like hierarchical structures, such as sheep and horses for animals, cars and trucks for motor vehicles, and both animals and motor vehicles belong to a superclass. Each category is represented by two parameters, mean and variance, and knowledge transfer between different categories is realized by transfer parameters. Literature [19, 20] put forward the application of twin networks to small sample problems, which opened the prelude of neural networks to deal with small sample problems. Literature [21, 22] also proposed probability matrix decomposition and Bayesian probability matrix decomposition. Literature [23] puts forward a recommendation algorithm, which can push hot topics personalized for users to solve the problem of information overload faced by users. The content-based recommendation is simple and intuitive, but limited by the description degree of project content, and the recommendation effect is not good. Literature [24] combines and uses a variety of machine learning methods to complete the classification of a large number of movie commentary corpuses. Literature [25] introduces new feature words of semantic role tagging and discusses the application effect of multifeature word combination mode through experiments. This method has some shortcomings. The manual tagging process will take a lot of time and manpower, and it is not suitable for large-scale corpus analysis and research.

## 3. Research Method

### 3.1. Online SSL Modeling

The set of fluctuating emotions generated by learners in the learning environment that accompanies the entire learning process is referred to as learning emotion. Detailing learners' emotional characteristics, particularly the classification of basic emotional types and specific emotional characteristics, is crucial to developing an online SSL model that incorporates healthy emotional factors. In an online classroom setting, the basic learning emotions are divided into six categories: happiness, surprise, boredom, sadness, fear, and anger. Emotions can be expressed in a variety of ways, the most intuitive and quickly recognized being a change in facial expression.

The learner model, which incorporates healthy emotional factors, is the foundation and key to online classroom learners' learning analysis. This chapter focuses on the study of learners' emotional characteristics, referring to the norms of learners' models and various learners' personalized models, and divides learners' characteristic models into learners' basic characteristics, behavioral factor characteristics, and emotional factor characteristics, based on conceptual combing and theoretical analysis. A learner model incorporating healthy emotional factors is formed based on the above research. Most learners' models are currently built by analyzing and mixing learners' models from a single dimension, and most studies focus on the characteristics of learners with behavioral factors, with little attention paid to the study of learners' emotional factors. However, as intelligent teaching evolves, more educators must consider the impact of learners' emotional states on learning outcomes, and the analysis of learners' emotional states is becoming a common concern in learning analysis.

**Figure 1:**
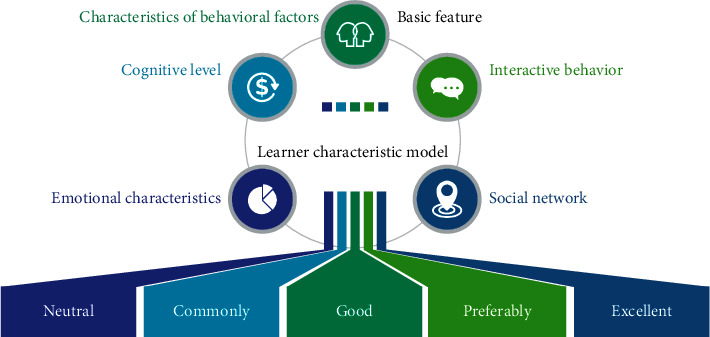
Online SSL model integrating health emotional factors.

In this chapter, on the basis of summarizing the learner model specification and referring to the learner's personalized model, an online SSL model integrating healthy emotional factors is proposed, as shown in Figure 1.

Social network features include dot centrality, feature vector, and reciprocity. Emotion features include emotion data collection, expression recognition, and basic learning emotion, and the model mainly shows the basic learning emotion features. The learner model integrating healthy emotional factors is the basis and key to analyze the diverse characteristics of learners in an online classroom environment.

To analyze learners' dynamic cognitive level, first, the learning behavior of learners is indexed and then judged whether these behavior indicators obey normal distribution. It can be analyzed with corresponding correlation analysis methods, and the significant correlation detection results are obtained and judged whether to predict the model or abandon the indicators. First, a learning behavior index set is established, and the learning behavior index set is set as *U*, then

(1) $$\begin{array}{c} U=\left\{u_{1},u_{2},\ldots u_{n}\right\}. \end{array}$$

Among them, *u*_*n*_ represents the learning behavior index, and *n* represents the number of learning behaviors. The goal of creating a learning behavior index set is to convert learners' specific learning behaviors into data and visual set elements, as well as to accurately present the cognitive level hidden behind learning behaviors so that learners' cognitive level can be accurately analyzed.

The data processed in SSL is often high-dimensional, and the distribution of data has no obvious structural information. Similarity *w*_*ij*_ between samples depends on the mapping function to map samples into a new space and then calculate *w*_*ij*_ in the new space. The specific calculation is as follows:

(2) $$\begin{array}{c} w_{ij}=\left\{\begin{array}{cc} e^{\frac{{\left\|f\left(x_{i}\right)-f\left(x_{j}\right)\right\|}^{2}}{2\sigma^{2}},} & i\neq j,\\ 0, & i=j. \end{array}\right. \end{array}$$

where *f* : *R*^*D*^⟶*R*^*M*^ represents a mapping function. *f* map all samples to an *M*-dimensional space.

Let the optimal solution of the objective function be the kernel matrix *K*^*∗*^. The next task is to infer the category of query samples from *K*^*∗*^. The category to which query $\hat{x}$ belongs is obtained by the following type of weighted voting:

(3) $$\begin{array}{c} \hat{y}=\arg\underset{i}{\max}\sum_{x_{j}\in s_{i}}k_{j}^{*}, \end{array}$$

where *s*_*i*_ represents the set of all support set samples of category *i*. In fact, the formula classifies the query samples into the category with the largest sum of kernel similarity, which is consistent with the classification idea in the matching network.

The frequency of course visits, the length of learning time, the degree of test completion, the number of data downloads, and other factors reflect the degree to which learners pay attention to education and reflect their behavior after accepting an online education platform. As a result, extracting and analyzing the characteristics of learners' learning behaviors are necessary in order to analyze learners' attitudes. To construct a learner's personalized model, relevant data from the database and behavior log data of an online education platform are extracted and mined. Personalized modeling, to put it simply, is the process of creating a detailed and personalized portrait of a learner.

**Figure 2:**
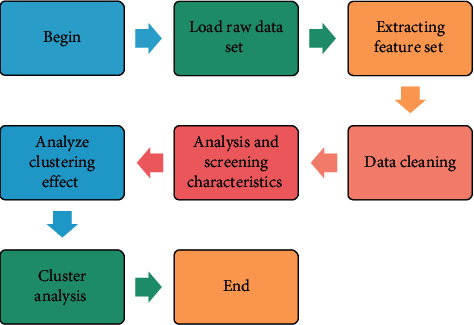
Generation flow chart of the multivariate cluster analysis model.

The specific steps of the learner attitude analysis model are shown in Figure 2. In this paper, both the original data set and the feature set related to learners' behaviors are continuous data by default.

To analyze learners' attitude model, data collection is first needed. The click data, watching video data, discussion area data, and test data of learners are extracted from the online education platform, and the data related to learners' learning behaviors are extracted from them.

### 3.2. Curriculum Recommendation Integrating Health Emotional Factors

Generally, universities offer many elective courses, and students' elective courses are limited to some extent, so it is easy for students' scoring data to be extremely sparse. Due to the extreme sparseness of student rating data, the traditional similarity measurement method cannot effectively calculate the nearest neighbor of the target user, and the quality of CFR (collaborative filtering recommendation) system is also difficult to guarantee. The key to construct the student rating data matrix is to solve the sparseness of the data set.

Teaching quality evaluation, as an integral part of the teaching management system, aids teachers and schools in significantly improving teaching quality. Teaching quality evaluation is the process of systematically collecting data and assigning a value to teaching activities and outcomes in the classroom based on the requirements of teaching objectives and principles. The main source of teaching feedback information is the evaluation of teachers' classroom teaching quality, and it is also an important way to test teaching effect and evaluate teaching quality.

Research shows that in many recommendation methods, the most critical “emotional intelligence” factor in communication between recommendation system and people is often ignored. Especially for specific courses, such as music and movies, emotional factors often play a decisive role in recommendation, which requires the recommendation system to be able to actively meet the emotional needs of students.

**Figure 3:**
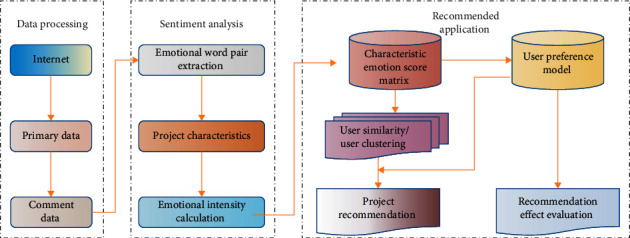
Personalized recommendation framework based on sentiment analysis.

The main idea is to pay attention to and mine the user comment data, mine the user's evaluation attitude towards each feature of the project, instead of calculating the user similarity based on the score information, measure the user's emotional similarity to the feature level of the project, and calculate the similarity and clustering by using the user's score of the feature of the project, so as to find the most similar *N* users for recommendation. The recommended framework is shown in Figure 3.

Content-based recommendation algorithm uses feature attributes to define course objects and makes recommendations based on students' evaluation of course objects. Therefore, the algorithm can be used to recommend courses in a sparse situation. First, the similarity between courses in the database and favorite courses is calculated, and formula (4)is used.

(4) $$\begin{array}{c} f\left(P,A\right)=\sum_{i=1}^{n}w_{i}\times \left(1-\left|p_{i}-\alpha_{i}\right|\right), \end{array}$$

where *P* represents a course in the database; *A* is a course that has been evaluated as a favorite by some students; *n* represents the number, of course, attributes corresponding to the category to which the course belongs; *w*_*i*_ represents the weight values of the above attributes; *m*_*i*_ represents the value of the *i*-th attribute of the course *P*; *α*_*i*_ represents the *i*-th attribute value of favorite course *A*.

By analyzing and calculating all the historical review data of a project, with the help of popular wisdom, it is possible to predict the scores of different attributes of the project.

(5) $$\begin{array}{c} {\bar{R}}_{k}=\frac{\sum_{i=1}^{N}s_{ik}}{N}, \end{array}$$

where *N* represents *N* comments under a specific course, and ${\bar{R}}_{k}$ represents the average emotional score of the specific course on the *k*-th feature.

Using natural language processing technology to analyze the comment texts in the check-in history records, and further explore the emotional tendency of the texts, can accurately understand users' preferences and further improve the recommendation quality of POI (point-of-interest) [12].

Text is composed of words, and the polarity of emotional words in comments represents the tendency of text, so the tendency of comments can be obtained by calculating the polarity of emotional words. There are many ways to calculate emotion, and PMI (point mutual information) method is the simpler and more effective one.

Emotional polarity calculation usually assumes that when a word has a strong correlation with positive words, it has positive emotional polarity and vice versa. The polarity of emotion words is determined by calculating PMI between target emotion words and terms in emotion vocabulary. The PMI value between words *w*_*i*_, *w*_*j*_ is defined as shown in formula (6).

(6) $$\begin{array}{c} PMI\left(w_{i},w_{j}\right)=\;\log_{2}\frac{p\left(w_{i},w_{j}\right)}{p\left(w_{i}\right)*p\left(w_{j}\right)}, \end{array}$$

where *p*(*w*_*i*_), *p*(*w*_*j*_) is the probability of *w*_*i*_, *w*_*j*_ in corpus, and *p*(*w*_*i*_, *w*_*j*_) is the probability of *w*_*i*_, *w*_*j*_ co-occurrence.

Friends often have similar behaviors and many common interests. Users usually refer to their opinions before visiting unknown POIs. Friends can influence users' choices of POIs more than ordinary users [14]. The distance between friends can be described by the number of the same friends among friends, and the similarity of interests among friends can be measured by the number of friends visiting the same POI. Therefore, the strength of the social relationship between users and their friends can be calculated by equation.

(7) $$\begin{array}{c} sr_{ij}=\frac{\left|F_{i}\cap F_{j}\right|}{\left|F_{i}\cup F_{j}\right|}*\frac{\left|L_{i}\cap L_{j}\right|}{\left|L_{i}\cup L_{j}\right|}, \end{array}$$

where *F*_*i*_, *F*_*j*_, respectively, represents the friend set of user *u*_*i*_, *u*_*j*_, and *L*_*i*_, *L*_*j*_, respectively, represents the sign-in POI set of user *u*_*i*_, *u*_*j*_.

Project recommendation generation is as follows:

(8) $$\begin{array}{c} R=\sum_{k=1}^{8}{\bar{R}}_{k}\times \lambda_{k}, \end{array}$$

where *λ*_*k*_ is the attention of the target user to the *k*-th feature, and *R* is the predicted score of the candidate project, which can be regarded as the recommendation degree.

## 4. Results Analysis and Discussion

### 4.1. Emotional Feature Analysis

In this paper, emotional analysis refers to the process of gathering user feedback on a product, analyzing the content of the feedback, determining the polarity of the user's emotional tendency at the feature level of the project, that is, the favorable or unfavorable attitude and opinion toward the course or project, and then extracting more useful information.

**Figure 4:**
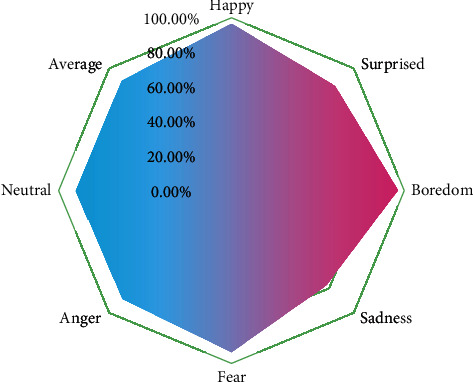
Recognition results of layered RF in mixed expression database.

According to the facial expression data of learners acquired by depth camera technology, the emotional state of learners is identified. The recognition result of the obtained layered RF (random forest) in the mixed expression database is shown in Figure 4.

Figure 4 shows that the hierarchical RF algorithm has a high recognition rate for learners' emotional state and can accurately judge learners' emotional state in the online classroom learning process.

**Figure 5:**
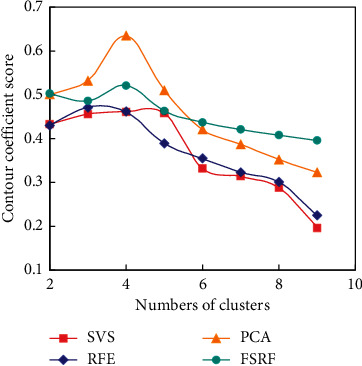
Influence of different feature selection methods on clustering effect.

It can be deduced from this that learners' emotional states changed during the stage of introducing the curriculum and teaching new lessons, and that learners always pay attention to the gradual progression of teaching content, and that learners' cognitive levels turn old and new, and that they actively construct meaningful knowledge. Features related to learners' learning behavior are extracted from all learners' features to form a feature set, which is used to analyze learners' learning attitude.  Figure 5 shows the comparison of clustering results using SVS (single variable selection), RFE (recursive feature elimination), PCA (principal component analysis), and FSRF (feature selection based on the random forest).

It can be seen that when the clustering is 4 and the feature selection method is PCA, the contour coefficient reaches the maximum, and the classification effect is the best. Finally, the learner's feature data set is divided into four categories; that is, the learner's attitude model is divided into four categories.

**Figure 6:**
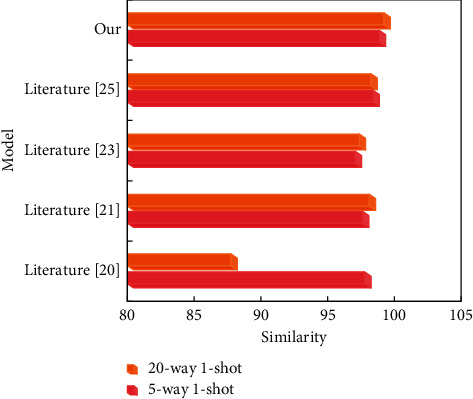
Small sample experimental results.

In order to evaluate the performance of the algorithm, a small sample experiment was carried out on two data sets, namely, Omniglot [16] and miniImageNet [18]. On Omniglot data set, this paper tests the performance of the algorithm under 5-way 1-shot and 20-way 1-shot tasks. The experimental results are shown in Figure 6.

It can be seen from the figure that the performance of the SSL method based on graph regularity is slightly worse than that of prototype network with interval, because when there is only one sample in each class, the relationship between classes cannot be propagated, and it is a good strategy to simply take this sample as the representative of this class.

### 4.2. Course Recommendation Analysis

Here, the criteria for evaluating recommendation quality are set as MER (mean error rate) and SV (standard variance). MER is to determine the recommendation quality by predicting the deviation between the category and the actual category. The higher the MER value, the worse the recommendation quality. The calculation of SV follows the statistical method, which can directly measure the recommended quality. Here, the content-based recommendation algorithm and CFR algorithm are compared, and the three algorithms are repeatedly executed 80 times, respectively, and the recommendation quality is shown inFigures 7 and 8:

The hybrid algorithm has higher recommendation accuracy and better stability than the other two recommendation algorithms, as shown in the above figure, and clearly improves recommendation accuracy. This paper designs and compares four simplified models of curriculum recommendation model incorporating healthy emotional factors, namely, GP, CPP, SI, and ET, in order to analyze the importance of GP (geographical position), CPP (classification popularity preference), SI (social intensity), and ET (emotional tendency) factors in POI recommendation in the curriculum recommendation model incorporating healthy emotional factors.

**Figure 9:**
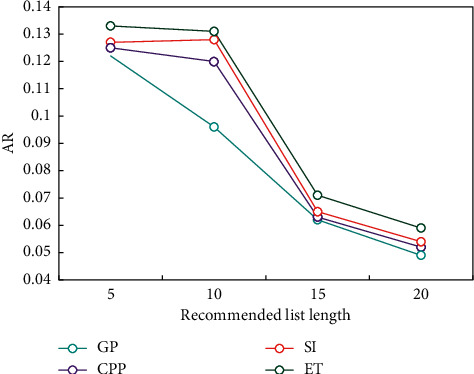
Comparison of different factors affecting recommended AR.

**Figure 10:**
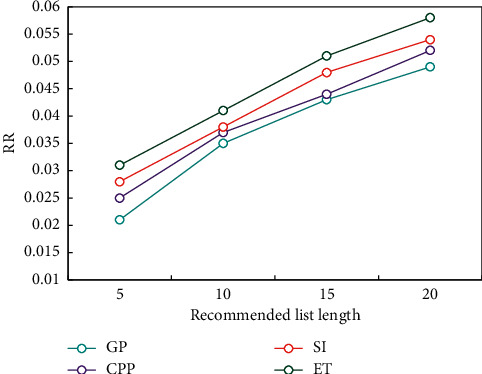
Comparison of recommended RR influenced by different factors.

As the evaluation indexes, AR (accuracy rate) and RR (recall rate) are chosen. The experimental results are shown in Figures 9 and 10.

Foreign countries have already integrated microcourses into daily teaching for students to preview and study independently, but domestic research on the application of microcourses is still relatively lagging behind, and the research on the process and effect analysis and evaluation of microcourses applied to teaching is not deep enough, and the research on the application of microcourses resources and learning experience tracking is almost blank. Because of the different teaching systems at home and abroad, the application of this model in China is still in the exploratory stage. Lessons from the successful experience of foreign applied microclass flipped classrooms are drawn, and the curriculum standards of various disciplines in our country are combined.

In this paper, the course features are clustered into 8 categories by analyzing the course evaluation set, and then, the attention of users to various attribute features is statistically obtained from the user's own comment set to form user preferences.

Traditional information literacy courses, like other courses, mostly use classroom teaching as the mainstay, supplemented by computer operation, and teachers as the mainstay and students as the supplement in the classroom, so it is difficult for students to reflect their personalized learning, and the teaching atmosphere can be imagined. Microcourses, on the other hand, have outstanding advantages, such as short time, abundant resources, and long online time, which meet the needs of students to flexibly choose learning time, learning place, and learning content to the maximum extent, and autonomous learning is relatively easy. At the same time, students can also reproduce the classroom teaching situation of a certain knowledge point at any time, which can well solve the problem of memorizing in class and forgetting after class, which is an important supplement and resource expansion of traditional classroom learning [7].

The key link in consolidating and digesting the curriculum's key and difficult problems is classroom teaching. Teachers break down teaching tasks and create various links to assist students in solving problems based on the syllabus. Teachers must periodically assess their students' knowledge and provide appropriate guidance and correction, as well as timely feedback. Teachers should be at the forefront of curriculum development, extracting a series of knowledge points from each class's teaching tasks based on the needs of class hours, and carefully designing and developing these knowledge points into special microcourses and series microcourses.

### 4.3. Teaching Suggestion

For starters, online education prevents students from interacting with teachers in real time, reduces teacher-student communication, fails to stimulate students' thinking, and causes them to lose their ability to think. Second, plagiarism in students' homework is a serious issue. Students are more likely to copy other people's learning achievements as the Internet has evolved, and they are unable to do so independently. Finally, the impact of paying attention in class has waned. After signing in before class, many students fall asleep and only watch the video, leaving the teacher to “perform” alone. To summarize, the quality of online education cannot be guaranteed at this time, and a good educational effect cannot be obtained in a holistic manner. This development raises concerns about the future of online education.

To digitize teaching, the online classroom makes use of big data and cloud computing technologies. It comprehensively assesses students' learning quality and efficiency, starting with daily classroom study, daily assessment, semester assessment, and other aspects, making teachers' evaluation of students more objective, accurate, and fair, making teachers accurately assess students' growth, making teachers' teaching more targeted, and improving classroom teaching quality and efficiency. Students can interact with teachers more effectively in an online classroom environment. Interactive activities such as sign-in, selection of students, competition for answers, and the combination of online and offline interaction greatly improve the interaction efficiency between teachers and students, which is conducive to students' autonomous learning and teachers' accurate grasp of students' learning situations.

Teachers in the new era should change their roles in time, from the leader of the traditional classroom to the leader of the online classroom, guide, organize, and coordinate students' learning activities, and cultivate students' autonomous learning and cooperative learning ability. The ability to master and apply modern information technology is a new test for teachers, which not only requires teachers to skillfully use all kinds of learning tools and software but also requires teachers to establish data awareness, master relevant data processing technologies, and digitize and visualize the teaching process, so as to more efficiently and accurately analyze the learning situation. To improve the information-based teaching quality, an information technology mutual assistance group is established, intragroup exchange and discussion activities are organized, and teachers are helped with weak information technology to improve their information technology teaching ability.

In the aspect of educational content, a student-centered educational philosophy is established, pay attention to the combination of theory and practice, the material needs of students are met, and their spiritual needs are emphasized; that is, a balance among objective knowledge, value, and morality is sought.

In terms of teachers, teachers should change their teaching role orientation in time according to the changes of the times, change the traditional knowledge transmitter to become an interactive learner, and change the teaching controller to become an activity leader. Only when teachers take the lead in the initial change, students can make progress.

## 5. Conclusion

Personalized learning research is based on the construction of the learner model. This study integrates the above research emphases. In view of the current situation of insufficient research on the characteristics of learners' emotional factors in the study and analysis of learner model, combined with online classroom teaching environment, online SSL model research integrating healthy emotional factors is carried out. Based on the proposed learner model, this paper studies the learner feature analysis technology, focusing on the learner's emotional feature analysis technology. In the experimental part, experiments of multiple tasks are carried out on data sets, which proves that SSL based on graph regularity is effective. According to the social relationship, the social intensity among users is calculated, and the emotional tendency of users according to the comment text is analyzed and effectively combined them with collaborative filtering recommendation technology, so as to get the social-emotional score. Through experiments, even under extreme test conditions, the algorithm still shows good recommendation accuracy and timeliness, which greatly improves the recommendation quality.

In the future work, we will further explore the influence of time factors and content information such as photos and videos generated by users in POI on users' sign-in behavior, so as to further improve the recommendation quality.

## Figures and Tables

**Figure 7:**
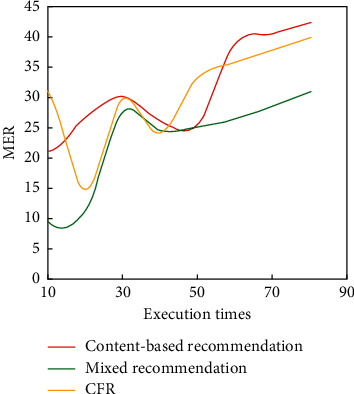
MER algorithm comparison.

**Figure 8:**
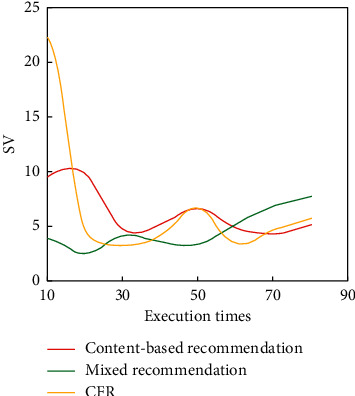
SV algorithm comparison.

## Data Availability

The data used to support the findings of this study are included within the article.
